# Case report: Tackling the complexities of an extremely premature newborn with intrauterine growth restriction and congenital metabolic disorders through a multidisciplinary approach

**DOI:** 10.3389/fped.2023.1162226

**Published:** 2023-06-19

**Authors:** Ioana Roșca, Andrei Gheorghe Preda, Andreea Teodora Constantin, Ciprian Coroleucă, Emilia Severin, Raluca Ioana Teleanu, Alina Turenschi

**Affiliations:** ^1^ Neonatology Department, Clinical Hospital of Obstetrics and Gynecology “Prof. Dr. P. Sârbu”, Bucharest, Romania; ^2^ Department of Pediatrics, Neonatology, and Specialized Care, Carol Davila University of Medicine and Pharmacy, Bucharest, Romania; ^3^ Pediatrics Department, National Institute for Mother and Child Health “Alessandrescu-Rusescu”, Bucharest, Romania; ^4^ Obstetrics and Gynecology Department, Clinical Hospital of Obstetrics and Gynecology “Prof. Dr. P. Sârbu”, Bucharest, Romania; ^5^ Department of Genetics, University of Medicine and Pharmacy “Carol Davila”, Bucharest, Romania; ^6^ Pediatrics Department, Children’s Clinical Hospital “Dr. Victor Gomoiu”, Bucharest, Romania; ^7^ Pediatrics Medical Resident, Department of Pediatrics, Carol Davila University of Medicine and Pharmacy, Bucharest, Romania

**Keywords:** extreme premature newborn, VLBW, hypoglycemia, L-carnitine deficiency, multidisciplinary approach

## Abstract

**Background and objectives:**

The premature birth of a newborn can present a complex challenge for healthcare providers, particularly in cases of extreme prematurity combined with intrauterine growth restriction and multiple metabolic deficiencies. In this report, we aim to shed light on the difficulties and considerations involved in the management of such a case. In addition, our study is aimed to raise awareness of the importance of a multidisciplinary team in managing an extreme premature case with multiple comorbidities.

**Case presentation and main findings:**

We present the case of a 28-week premature female newborn with very low birth weight (660 g, percentile <10%) and intrauterine growth restriction. She was born through emergency cesarean delivery due to maternal Hemolysis, Elevated Liver enzymes, and Low Platelet count (HELLP) syndrome and had a high-risk pregnancy (spontaneous twin pregnancy, with one fetus stopping development at 16 weeks and maternal hypertension). In the first hours of life, she presented with persistent hypoglycemia requiring progressive glucose supplementation up to 16 g/kg/day to maintain normal blood glucose levels. The baby then showed favorable progress. However, from days 24 to 25, hypoglycemia recurred and did not respond to glucose boluses or supplementation in both intravenous and oral feeds, leading to the suspicion of a congenital metabolic disorder. Endocrine and metabolic screenings led to suspicion of primary carnitine deficiency and a deficiency in hepatic form of carnitine-palmitoyltransferase type I (CPT1) on the second screening.

**Conclusion and clinical implications:**

The study highlights rare metabolic anomalies that can be due to both organ and system immaturity and delayed enteral feeding and excessive use of antibiotics. The clinical implications of this study emphasize the need for careful monitoring and comprehensive care of premature infants to prevent and manage potential metabolic abnormalities by neonatal metabolic screening.

## Introduction

Prematurity is a major global public health issue, with significant impacts on neonatal morbidity and mortality. The incidence in Romania is around 10%, making it the leading cause of neonatal death. Advances in technology have made it possible for extremely premature infants with low birth weights to survive, but this has resulted in difficulties in managing complex cases (1). Premature infants are at risk of developing carnitine deficiency due to their immaturity and their increased metabolic demands. L-carnitine plays a crucial role in the oxidation of fatty acids, especially during the neonatal period. While breast milk and formula milk contain carnitine, it is missing in most parenteral nutrition solutions (2). The carnitine content in total parenteral nutrition (TPN) solutions is often low, which can lead to a deficiency of this essential nutrient. Early carnitine supplementation has been shown to be effective in preventing and treating carnitine deficiency in premature infants receiving TPN. The clinical implications of carnitine deficiency in premature infants receiving TPN include an increased risk of hypoglycemia, insulin resistance, and liver dysfunction. In severe cases, carnitine deficiency can lead to cardiomyopathy and neurodevelopmental delays. The management of carnitine deficiency in premature infants receiving TPN requires a multidisciplinary approach, including close monitoring of the infant’s metabolic status, early detection of deficiency, and prompt initiation of carnitine supplementation.

## Case presentation

We report the case of a premature very low birth weight (VLBW) newborn, with 28 weeks’ gestation, with intrauterine growth restriction (IUGR), weighing 660 g at birth (percentile <10%), Caucasian female, from a high-risk pregnancy.

The parents of the child are non-consanguineous and have no significant medical history. The mother was 29 years old (Gravida I, Para I), when she spontaneously conceived a twin pregnancy. The father was 28 years old and had type II diabetes and hypertension. The maternal grandmother has a history of mixed connective tissue disease.

Initially, the pregnancy was a twin pregnancy, with one fetus stopping its development at 16 weeks, and the surviving twin presented intrauterine growth restriction, being delivered by cesarean section at 28 weeks. Other pregnancy complications included gestational hypertension (169/100 mmHg) that developed after the first twin stopped developing. Gestational HTA (hypertension (high blood pressure)) was managed by medication, such as Dopegyt, Adalat, Clexane (4,000 UI–0.4 ml/day between 16 and 28 weeks of gestation), and Aspenter (75 mg × 2/day from week 12 to 24 of gestation).

The patient also experienced nausea and sleepiness throughout the pregnancy. Prenatal care was done by a multidisciplinary team formed by the family doctor, obstetrician, and cardiologist. The baby was extracted by emergency cesarean section due to maternal Hemolysis, Elevated Liver enzymes, and Low Platelet count (HELLP) syndrome from a cephalic presentation.

## Baby's condition shortly after delivery

The newborn presented with a severe general condition at birth, diffuse generalized cyanosis, normal umbilical cord insertion, white subcutaneous tissue, two arteries and one vein, rhythmic heart sounds without murmurs, heart rate >100 beats/min, spontaneous but shallow and inefficient breathing, decreased tone and reactivity, supple abdomen, patent anus, and no clinically detectable malformations. The Apgar score was 4 at 1 min, 6 at 5 min, and 7 at 10 min. Considering the inefficient breathing with generalized cyanosis, extremely low gestational age and weight, it was decided to perform orotracheal intubation with prophylactic surfactant administration (200 mg/kg/dose) and alveolar recruitment using the Neo-Puff system during the first 30 min of life. The newborn's color improved after 30 s of ventilation, becoming pink and with SpO_2_ >95% (FiO_2_ 30%) at 10 min of life. The newborn was admitted to the neonatal intensive care unit, placed at the thermal neutral point, and intubated and mechanically ventilated in the Synchronized Mandatory Intermittent Ventilation (SIMV) system for 37 days, and then extubated and ventilated in the Continuous Positive Airway Pressure (CPAP) system for 3 days.

## Baby's condition in the first hours of life

**Figure 1:**
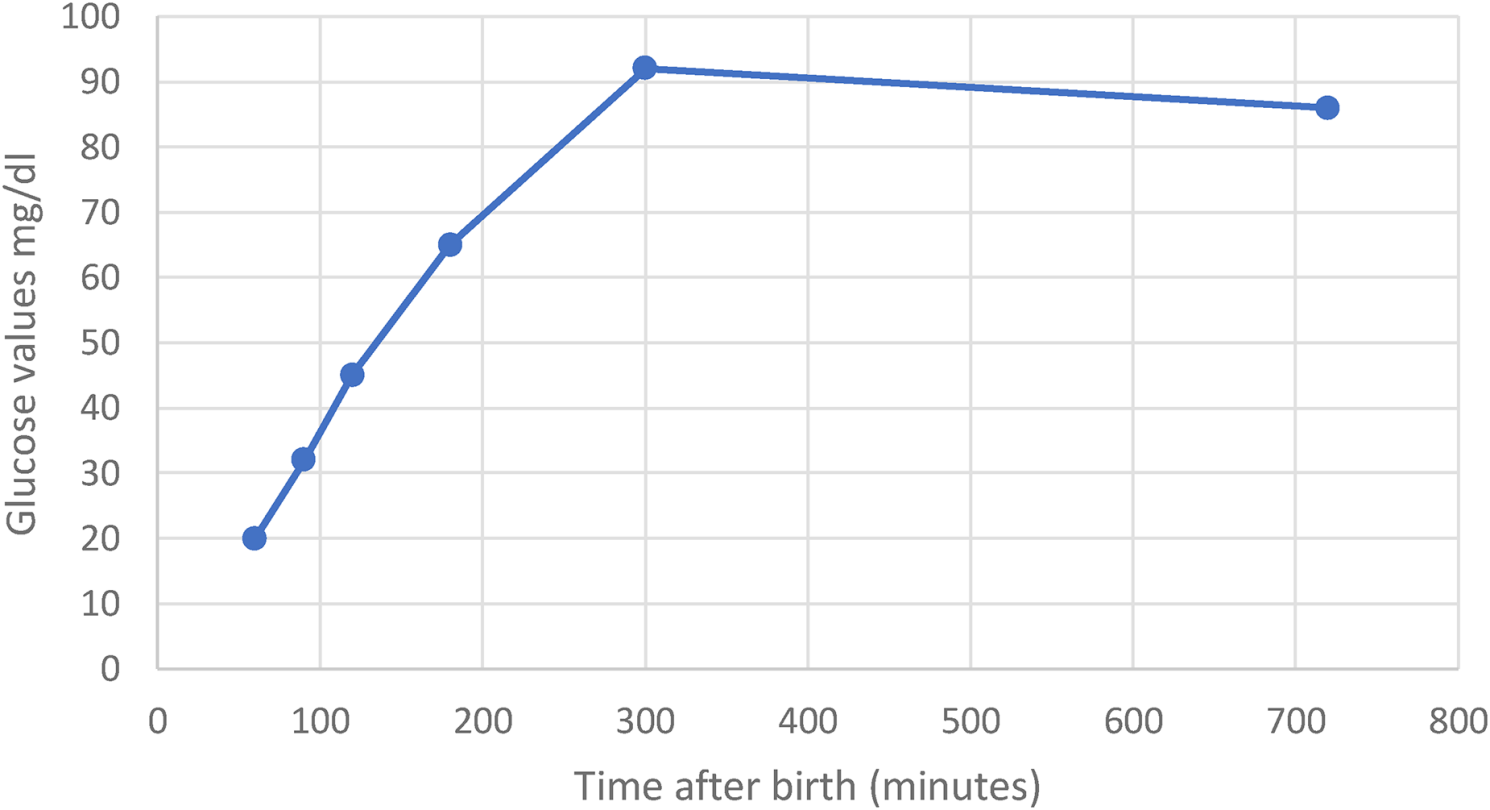
Glycemic monitoring in the high-risk newborn.

The newborn presented with neonatal hypoglycemia from the first hours of life (blood glucose on a glucometer = 20 mg/dl at 1 hour of age, 32 mg/dl at 1 h 30 min, 45 mg/dl at 2 h, and 60 mg/dl at 3 h) which was corrected by increasing the IV glucose amount according to the guideline, later with a favorable evolution (Figure 1).

## Baby's condition in the first month of life

**Figure 2:**
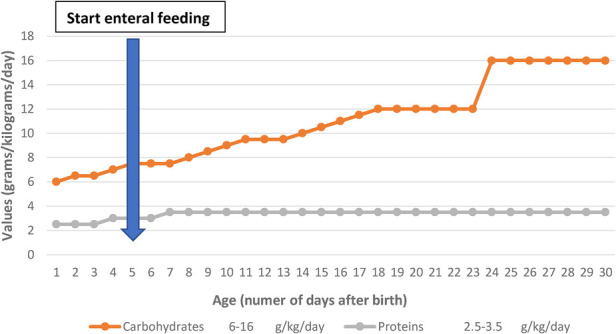
Evolution of protein and carbohydrate requirements in the first 30 days of life and enteral feeding started on day 5 of life.

On days 24–25 of life, the newborn presented with clinical lethargy, hypotonia, cyanosis with increased ventilation parameters, feeding difficulties, and paraclinical blood glucose 22 mg/dl, persistent hypoglycemia that does not resolve with glucose bolus administration and supplementation in perfusion requiring increased glucose intake of 16 g/kg/day (Figure 2), and hydrocortisone hemisuccinate was added to the treatment (used as part of therapy) to maintain normal glycemia, for which a congenital metabolic disease is suspected. The newborn maintained normal blood glucose values under this treatment.

Endocrine and metabolic screening resulted in a suspicion of carnitine deficiency, second screening, and CPT1 deficiency—the hepatic form (carnitine palmitoyltransferase type I). Hemisuccinate hydrocortisone was introduced for treatment to maintain normal glycemia. Throughout the evolution, the newborn presented with numerous acute and chronic complications of prematurity requiring follow-up and interdisciplinary consultations. The newborn presented with maternal-fetal infection and later with repeated urinary infections requiring prolonged antibiotic therapy.

The patient required prolonged mechanical ventilation (41 days), later being oxygen-dependent for 30 days, requiring FiO_2_ 30%–40% to maintain SpO_2_ >95%, thus being diagnosed with bronchopulmonary dysplasia, for which corticotherapy, diuretics, and azithromycin were prescribed with a favorable evolution, maintaining SpO2 >95% in atmospheric air.

Considering the extreme prematurity, the prematurity apnea profiling was performed with intravenous caffeine for 67 days (until the corrected age of 37 weeks).

From a cardiovascular point of view, the newborn was stable throughout the hospitalization, but cardiology consultations and echocardiograms were performed (biventricular hypertrophy in remission, patent foramen ovale), knowing the risks of these extreme preterm infants.

**Figure 3:**
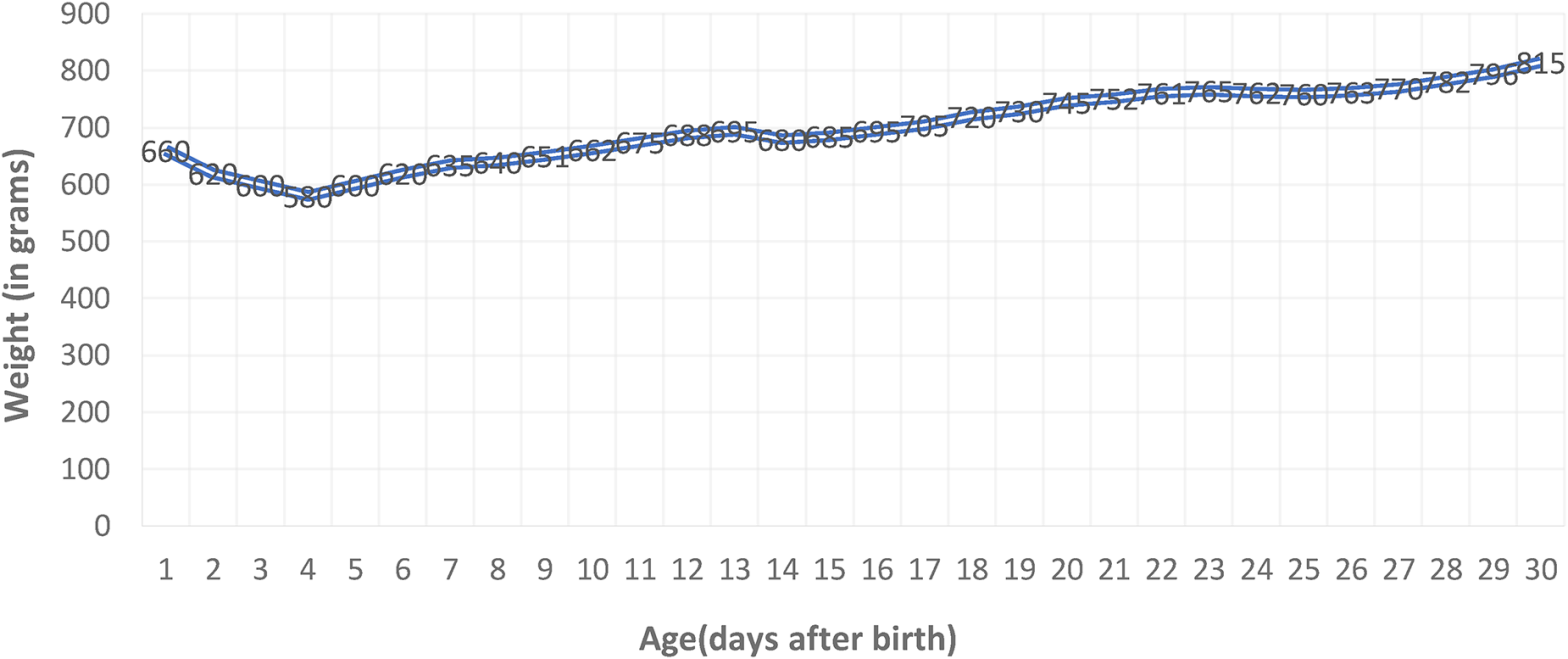
Preterm weight curve: slow ascent with a weight loss of 12% corresponding to the age, followed by stagnation on days 23–25 with decreased digestive tolerance, and then with a favorable evolution.

Being a premature newborn with intrauterine growth restriction and hemodynamic instability, enteral feeding was temporarily postponed in the first 5 days of life (exclusively parenteral nutrition), and later fed with a hydrolyzed milk formula by discontinuous gavage in small quantities due to poor digestive tolerance, with a slow weight gain curve (Figure 3). The weight curve was slow in ascension during the first month of life, with a plateau in days 23–25, but later improved.

The patient also presented with aggressive retinopathy of prematurity, requiring bevacizumab injection.

Other complications associated with prematurity that arose during the hospitalization should also be mentioned: multi-etiology anemia (prematurity, iatrogenic—repeated collections, infections), for which repeated red blood cell transfusions were performed—16 administrations during hospitalization, multi-etiology jaundice, umbilical hernia, gastroesophageal reflux, and delayed neuromotor development.

Table 1 shows the laboratory investigation results, based on which the patient monitoring was conducted.

**Table 1:** Newborn monitoring sheet: case of a premature VLBW newborn.

Date	19.09	20.09	22.09	23.09	26.09	28.09	05.10	10.10	13.10	17.10	27.10	07.11	14.11
General hematology													
Blood group/Rh	O+												
Leukocytes, 10^3^/mmc	13.48		9.98		15.46	16.86	15.43	15.31		12.96	14.40	17.41	9.54
Neutrophil, %	9.30		32.70		58.80	49.50	52.10	57.00		65.90	73.30	52.20	45.10
I/T (percentage of immature granulocytes), %	0.10		2.20		5.40	5.60	2.30	2.30		1.50	1.50	4.60	1.20
Hemoglobin, g/dl	13.00		10.20		13.00	11.40	13.90	10.30		11.80	12.30	10.90	12.80
Hematocrit, %	41.60		32.20		39.50	34.00	40.70	30.60		34.00	35.30	31.30	37.50
Platelet count, 10^3^/mmc	164.00		257.00		341.00	258.00	581.00	408.00		635.00	334.00	742.00	353.00
General biochemistry													
Alk-phosphatase, iu/L											235		
ALT, iu/L		9.00		10.00						16.00	19.00		29.00
AST, iu/L		55.00		39.00						43.00	45.00		46.00
LDH, iu/L		1,490.00		1,424.00						1,040.00	884.00		
GGT, iu/L											903.52		
Creatinine, mg/dl		0.73		0.62						0.39			
Urea, mg/dl		48.20		41.45						18.96			
CRP, mg/L	0.62	0.35		0.08		0.01		0.03		0.03		0.02	
Total bilirubin, mg/dl		6.12		7.09									
Direct bilirubin, mg/dl		0.65		0.87									
Total protein, g/dl										4.63			
Bacteriological tests													
Urine culture									<1,000 UFC/ml				<1,000 UFC/ml
Blood culture	Negative at 7 days												

Date	17.11	22.11	28.11	05.12	15.12	23.12	28.12	29.12	06.01	12.01
General hematology										
Leukocytes, 10^3^/mmc	10.00	6.53	7.09	7.80	13.71	7.89		7.69	6.59	8.15
Neutrophil, %	42.60	38.20	41.10	32.00	64.20	36.60		40.10	30.00	31.40
I/T (percentage of immature granulocytes), %	1.40	1.20	1.40	1.30	1.00	0.50		0.30	0.20	0.10
Hemoglobin, g/dl	12.00	14.70	12.20	9.90	10.70	12.70		13.40	12.20	14.40
Hematocrit, %	34.50	43.40	35.90	29.10	31.40	37.80		40.10	35.70	42.50
Platelet count, 10^3^/mmc	251.00	315.00	382.00	386.00	610.00	568.00		491.00	603.00	490.00
General biochemistry										
ALT, u/L		31.00	27.00	25.00	22.00	44.00		41.00	30.00	30.00
AST, u/L		57.00	55.00	45.00	31.00	72.00		47.00	57.00	54.00
LDH, u/L	819.00	895.00	680.00	706.00	721.00	1,338.00		686.00	742.00	843.00
Amylase, u/L										
GGT, u/L										
Creatinine mg/dl	0.34	0.29	0.19	0.30	0.32	0.25		0.26	0.26	0.32
Urea mg/dl	21.77	17.76	16.94	23.79	47.96	23.06		36.86	20.42	25.53
CRP, mg/dl	0.06	0.05	0.09	0.06	0.03	0.02		0.04	0.04	0.01
Bilirubin total, mg/dl								0.62		
Bilirubin direct, mg/dl								0.18		
Protein (total), g/dl			3.95	4.19				4.87	4.64	
Urine culture			∼50,000 UFC/ml *Escherichia coli* strain produces beta-lactamase					>100,000 UFC/ml *Enterococcus faecium*		

VLBW, very low birth weight; I/T ratio, Immature to Total neutrophil ratio; ALT, Alanine Aminotransferase; AST, Aspartate Aminotransferase; LDH, Lactate Dehydrogenase; GGT, Gamma-Glutamyl Transferase; CRP, C-Reactive Protein.

## Baby's condition at discharge from the maternity ward

Upon discharge from the maternity ward at 4 months of chronological age, the baby's condition was good. The baby had pink integuments, an umbilical hernia, was breathing spontaneously and efficiently without any difficulty, and had SpO_2_ levels greater than 95% in atmospheric air without any desaturations. The baby was taking bottle feedings well, particularly the anti-reflux formula. The baby’s tone and reactivity corresponded to the corrected age, and she had an upward weight curve. The weight on discharge was 2,200 g.

## Follow-up and monitoring—the continued observation and care of the baby after discharge from the hospital

The baby's progress at home showed improvement in respiratory, neurological, and cardiac health, but there was minimal weight gain. As a result of extrauterine growth restriction, the baby required hospitalization in the pediatrics department. Genetic and endocrine consultations were repeated, and the results of the blood tests were within the normal range.

## Discussion

The complications of prematurity affect most of the organs and systems of the body due to their immaturity; hypoglycemia is one of the most frequent changes in homeostasis found in preemies due to increased energy needs, low glycogen deposits, and reduced gluconeogenesis. The etiology of hypoglycemia in preemies is often complex. In extreme premature cases, it is associated with intrauterine growth restriction (chronic fetal distress), perinatal hypoxia (acute distress), digestive rest, and risk factors for hypoglycemia (3). Other causes of hypoglycemia are secondary to the following perinatal stress conditions: sepsis, asphyxia, post-resuscitation, and respiratory distress (4, 5), and the presented case meets these conditions. The premature infant with IUGR requires neonatal screening for hypoglycemia immediately after birth from umbilical cord blood as they have two risk factors (prematurity and IUGR) for developing hypoglycemia (6, 7).

Persistent hypoglycemia and lack of correction by intravenous supplementation have raised suspicions of a metabolic disorder, which is why an endocrine and metabolic screening was performed, collecting insulin, peptide C, free fatty acids, growth hormone, beta-hydroxybutyrate, Adrenocorticotropic Hormone (ACTH), cortisol, and acyl carnitine from urine, with results within normal limits. A second metabolic screening was performed because of the oscillating clinical evolution that raised suspicions of carnitine deficiency and CPT1 deficit—the hepatic form (carnitine-palmitoyltransferase type I), a result that can be falsely positive in the case of an extremely premature, long-treated with antibiotics and steroids, as in the presented case, total parenteral nutrition, and gastrointestinal and renal disorders that are specific to preemies. Total parenteral nutrition is given to any extremely low birth weight premature, but the initiation of trophic enteral nutrition is essential for their future development, depending on the child's condition, comorbidities, and the specialist's experience. It has been shown that, in the case of premature infants, the administration of trophic nutrition leads to a significant increase in hormonal levels: enteroglucagon, gastrin, gastric inhibitory peptide, motilin, and neurotensin; in addition, increased levels of intestinal lactase and a reduction in intestinal permeability have been demonstrated at 10 days of life (7). Since these preemies have low immunity and come from pregnancies with increased risk of maternal-fetal infection, antibiotic therapy is necessary, but it should not be given in excess and should reduce both the number of antibiotics administered and the duration of treatment.

Carnitine is an amino acid with an important role in the transport of fatty acids to the mitochondria, where beta-oxidation occurs (8). The plasma and tissue levels of carnitine are low during the neonatal period (9). Although formula and breast milk contain carnitine, it is missing from parenteral nutrition formulas. Preemies are at risk of deficiency due to the immaturity of synthesis systems, delayed oral feeding, and increased urinary elimination (10). Currently, there are no data in the literature that indicate routine supplementation with carnitine for all premature infants (11).

Recent data show that supplementation with carnitine in parenteral nutrition solutions increases serum carnitine levels, but no improvement has been observed in lipid profile or mortality (12). There are also studies that show that low carnitine levels can be an etiological factor for the ability of premature infants to utilize intravenous lipids, as was the case with our patient. Hydrocortisone hemisuccinate treatment was instituted for the treatment of two pathologies: refractory/persistent hypoglycemia and bronchopulmonary dysplasia, with a positive result for both pathologies.

The pregnancy evolution was difficult, with one of the fetus's ceasing developments and the pregnancy being complicated with gestational hypertension. Despite medication administered to the mother, the surviving twin presented fetal distress leading to intrauterine growth restriction. Managing an extremely premature baby with growth restriction is much more complex and challenging than managing a premature baby without fetal distress. The VLBW newborn showed an oscillating evolution in the maternity ward, with multiple complications of prematurity, both acute and chronic, worsened by severe hypoglycemias that were difficult to manage but solved through good interdisciplinary communication (neonatologist, endocrinologist, pediatrician, geneticist).

In the case of carnitine palmitoyltransferase 1A (CPT1A) deficiency, hypoglycemia must be prevented, infants should eat frequently during the day and have cornstarch continuously at night, and fasting should not last more than 12 h during illness, surgery, or medical procedures, so prolonged fasting should be avoided (13). Individuals with CPT1A deficiency should have testing of liver enzymes (Aspartate Aminotransferase, Alanine Aminotransferase, alkaline phosphatase) and liver function (including Prothrombin Time and Partial Thromboplastin Time) at clinic appointments, even when asymptomatic, and during periods of reduced caloric intake (13). These tests were performed in our case and were within normal limits.

Critical hypoketotic hypoglycemia is a common presentation with most patients having recurrent hypoglycemic events as the first manifestation especially in the event of prolonged fasting or infection where glycogen reserves are exhausted soon (14, 15). In the present case, the patient had a lot of hypoglycemic events immediately after birth and at 3 weeks of life during which he presented a difficult digestive tolerance and serious general condition being mechanically ventilated, parenteral nutrition, and antibiotic treatment for infection. Key to management is in prevention of hypoglycemia. A sufficient level of glucose should be constantly supplied to patients with CPT1A deficiency during long time of fasting. Higher glucose infusion rates are essential in CPT1A-deficient patients along with mandatory blood glucose level monitoring during stressful conditions including major operation and severe infection (14, 15). We increased the level of IV glucose at maximum to prevent complications of hypoglycemia, especially neurological sequelae.

Inborn errors of metabolism present nonspecific clinical signs and may include decreased activity, lethargy, poor feeding, vomiting, respiratory distress, or seizures. These signs are common to several other neonatal conditions, such as sepsis and cardiopulmonary dysfunction (4). Our case was a preterm who presented a lot of these signs that are specific to the pathology of premature infants including hypoglycemia. It was so difficult to delimit the border between hypoglycemia due to prematurity and IUGR from hypoglycemia due to metabolic diseases. The results of the newborn screening test may be unreliable if the infant has received a blood transfusion; the clinician should repeat a newborn screening test at approximately 2–3 days after the last transfusion (5). In our case we respected it.

CPT1A deficiency is inherited in an autosomal recessive manner and requires genetic counseling. Heterozygotes (carriers) are asymptomatic, although heterozygous pregnant women may be at risk of developing acute fatty liver of pregnancy if the fetus has CPT1A deficiency (13).

## Conclusions

The survival and favorable development of extremely premature infants relies heavily on multidisciplinary consultations, both in the womb and during their extended hospital stay (4–5 months) and after discharge in the first years of life. The success of these cases is also due to medical policies and programs, as well as state-of-the-art equipment in maternity hospitals.

We consider that these high-risk pregnancies should benefit from hospitalization and multidisciplinary/interdisciplinary consultations in a high-level maternity ward capable of managing such cases (including hematology), the convening of a medical commission (obstetrician–neonatologist), and the completion of the pregnancy in an optimal time, so as not to prolong fetal distress leading to neonatal complications. In neonatology, there should be more courage in initiating trophic feeding, knowing its benefits, as early as possible (within the first 48 h of life), and avoiding the use of long-term antibiotic therapy.

As a takeaway message, in difficult pregnancies with multiple complications and a premature birth with intrauterine growth restriction, it is important to anticipate a difficult and fluctuating postnatal evolution. To improve the outcome, it is recommended to avoid prolonged fasting, long-term total parenteral nutrition, and long-term antibiotic therapy.

Despite their severe condition and multiple complications due to extreme prematurity and growth restriction occurring in the first and second trimesters of pregnancy, the newborn was discharged from the hospital to their home after 4 months of chronological age and was cared for by responsible parents who continue to follow the recommended interdisciplinary consultations. However, there is a lack of follow-up programs for premature infants in our country.

## Data Availability

The raw data supporting the conclusions of this article will be made available by the authors, without undue reservation.
